# Defining expert opinion in clinical guidelines: insights from 98 scientific societies – a methodological study

**DOI:** 10.1186/s12874-025-02534-0

**Published:** 2025-04-02

**Authors:** Blin Nagavci, Zsófia Gáspár, Botond Lakatos

**Affiliations:** 1 Doctoral School of Clinical Medicine, Semmelweis University, Budapest, H-1097 Hungary; 2 National Institute of Hematology and Infectious Diseases, Central Hospital of Southern Pest, Budapest, H-1097 Hungary; 3 Departmental Group of Infectious Diseases, Department of Internal Medicine and Hematology, Semmelweis University, Budapest, H-1097 Hungary

**Keywords:** Guidelines, Expert opinion, Methods, Society, Recommendations, Evidence, Transparency

## Abstract

**Background:**

The use of Expert Opinion (EO) in clinical guidelines is highly variable and lacks standardization, leading to ongoing controversy. A clear and universally accepted definition of EO is also lacking. To date, no research has systematically assessed how guideline-developing societies conceptualize and apply EO. This study aims to map methodological manuals, evaluate their rationale for EO use, examine its foundations, and synthesize a comprehensive definition.

**Methods:**

Systematic searches for clinical guidelines were conducted in PubMed to identify guideline-developing societies, supplemented by additional searches. Systematic searches were then conducted to identify methodological manuals from these societies. Screening was performed independently by two reviewers, and data extraction was conducted using piloted forms. Findings were summarized through narrative evidence synthesis using descriptive statistics.

**Results:**

A total of 473 national and international societies were identified, and methodological manuals from 98 societies were mapped and analysed. These manuals included 61 handbooks, 29 journal articles, and 8 websites. EO is mentioned in 65 (66%) manuals, with substantial variation in its utilization and terminology. EO is primarily used in two contexts: (1) filling evidence gaps (72%), and (2) interpreting existing evidence (8%). In the remaining 20%, EO use is unclear. Five main foundations could be identified as a potential basis for EO (clinical experience, indirect evidence, low-quality evidence, mechanism-based reasoning, and expert evidence/witnesses). Based on these findings, a novel comprehensive definition of EO was synthesized.

**Conclusions:**

EO is widely used to address evidence gaps and interpret ambiguous evidence, underscoring its importance in guideline development. However, the variability in its application and conceptualization across societies highlights the need for standardization. We propose a comprehensive EO definition as a first step towards standardization to improve consistency, transparency, and clinical decision-making.

**Supplementary Information:**

The online version contains supplementary material available at 10.1186/s12874-025-02534-0.

## Contributions to the literature

- The findings of this study highlight the need to bridge the divide between two opposing perspectives: one that entirely dismisses EO in guideline development and another that utilizes it without defined criteria.
- We advocate for a harmonized framework to standardize the application of EO in guidelines, tailored to the practical needs of guideline-developing societies.
- This study, along with the proposed definition of EO, offers a foundation for developing such standardization frameworks.

## Background

Clinical guidelines are essential for guiding clinicians and standardizing care. To be effective, they must address clinically relevant issues while adhering to robust methodological standards [1]. Despite significant improvements in guideline quality over time, challenges persist, particularly regarding the unclear utilization of expert opinion (EO) and evidence in guideline development process [2–5]. Guideline developers often rely on EO to formulate recommendations, in cases where high-quality evidence is unavailable, as shown by our previous work where half of infectious disease guidelines published between 2018 and 2023 permitted the use of EO [4]. In the era of evidence-based medicine, this practice remains a controversial topic, largely because it is not well regulated. Some experts argue that EO should be separated from evidence and not form the basis for recommendations [6, 7]. In contrast, others view EO as an aggregation of knowledge derived from diverse sources, which can be invaluable for guideline development [8, 9]. These differences are reflected also in the guideline development process, with large variations between them in how they utilize EO [2].

A key aspect to this problem, is the absence of a widely accepted definition of what EO is and what is represents. Until now, there is no research available on how guideline-developing societies conceptualize and address EO at the level of their methodological manuals. As these documents are the foundational frameworks for guideline development, they represent a critical unit of analysis for exploring and understanding the concept of EO. Addressing this critical gap could clarify definitions, rationales, and methodologies employed by the societies. This will facilitate development of standardized approaches for utilization of EO, aiming at reducing inconsistencies within and between guideline-developing societies and maximising transparency.

This study aims to: 1) systematically map methodological manuals from national and international guideline-developing societies, 2) evaluate the rationale and scenarios for EO use, 3) examine the foundations and origin of EO, and 4) synthesize a clear, standardized definition of EO based on data.

## Methods

This methodological study was reported in accordance with guidelines for reporting meta-epidemiological research [10, 11]. An internal protocol was developed to ensure methodological consistency and robustness (Additional file 1).

### Literature searches and screening

A systematic three step process was used to identify methodological manuals, by searching for clinical guidelines, identifying guideline-developing societies, and searching for methodological manuals from those societies:

#### Step 1) Systematic literature searches for clinical guidelines

Systematic searches were conducted in PubMed for clinical guidelines published between 2019 and March 2024 in all fields of medicine, using MeSH terms and keywords (Additional file 1). This time frame was chosen because only one guideline was required to identify a society, and older guidelines were unlikely to add further relevant information.

The retrieved references were screened in two phases, title/abstract screening and full-text screening. The screening was done in a Rayyan, by a single reviewer as the inclusion criteria were broad and the process was quite straight forward. Clinical guidelines published by national or international societies or organizations were deemed relevant. Guidelines published in English or with English abstracts were included, and the most recent versions were selected in case of multiple updates. Exclusion criteria included guidelines on dentistry, psychology and psychiatry, as well as technical documents such as health technology assessments. Guidelines not published in English were also excluded. Screening results, along with reasons for exclusion, are presented using the Preferred Reporting Items for Systematic Reviews and Meta-Analyses (PRISMA) flow diagram [12].

#### Step 2) Identification of societies

The included clinical guidelines identified in Step 1 were checked to identify all societies, organizations, groups of authors, or initiatives mentioned either as developers of the guideline or as endorsing societies. For each guideline the title, the background and the methods section were checked to extract society names. The identified societies and organisations were listed in Excel. This was supplemented with societies identified from previous work [4, 13, 14], and through additional hand searches in Google. The pooled list was then deduplicated to ensure that only unique national and international medical societies were included. All national or international non-governmental or governmental societies or organisations were considered relevant. Working groups of authors or initiatives outside formal societies were excluded from further analysis.

#### Step 3) Searching of methodological manuals

Finally, for each included society, systematic searches were performed to identify methodological manuals outlining their approach to clinical guideline development. As this was a more complex process, two reviewers independently searched for relevant documents on all official society websites. In addition, Google advanced search was also used with a predefined search string with multiple keywords (Additional file 1). All retrieved documents were then screened by two reviewers independently. Discrepancies between reviewers were resolved by discussion or through consultation with a third reviewer. Methodological manuals were included if they met the following criteria: 1) Any document, webpage, or article addressing the methodology for clinical guideline development of a society, 2) Published in English, and 3) Published from 2010 onward (most recent version was selected if multiple documents were available). Screening results, along with reasons for exclusion, are presented using PRISMA flow diagrams [12].

### Data extraction and analysis

Data from the included methodological manuals was extracted using piloted forms in Microsoft Excel, by a single reviewer and checked by another. Any discrepancy was resolved by discussion or involving a third reviewer. A narrative evidence synthesis was conducted to summarize the findings, employing descriptive statistics, using means and frequencies to summarize relevant data. Data was analysed in Microsoft Excel by a single reviewer and checked by another, with any disagreements resolved by discussion. List of extracted variables is available in the protocol (Additional file 1).

ChatGPT (version 4) was used as an assistive tool for spelling checks and improving text flow, using the prompt: 'Check text’s structure and flow.' AI was not used for data extraction, analysis, or interpretation. All AI-generated suggestions were critically reviewed and edited by the authors.

## Results

**Fig. 1:**
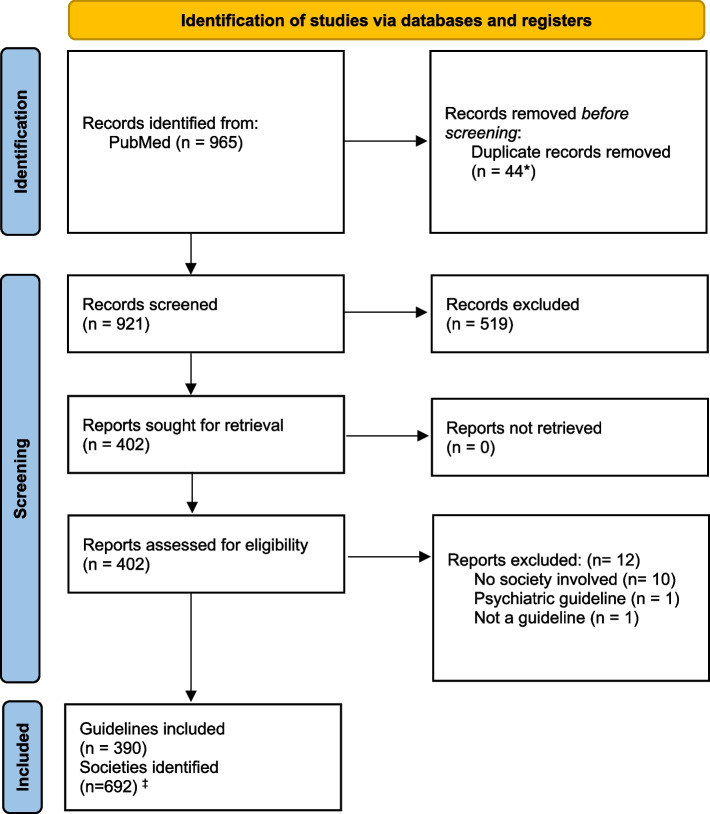
PRISMA flow diagram: Screening of Clinical Guidelines. *Due to clinical guidelines published in two journals simultaneously. ‡ Before de-duplication

**Fig. 2:**
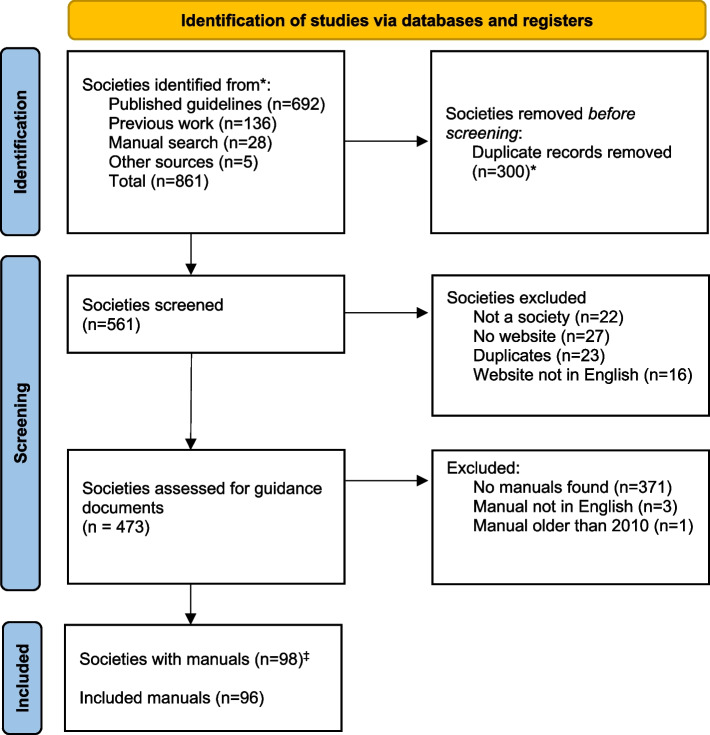
PRISMA flow diagram: Screening of methodological manuals. *Due to multiple search strategies and instances where the same guideline was published by multiple societies. ‡Two cases where societies use the same manual

The systematic literature searches for clinical guidelines identified 965 references, which were screened by title and abstract. Among these, 402 guidelines were selected for full-text review, and 390 were deemed relevant and included (Fig. 1). From these guidelines, 692 societies were identified and listed. Additionally, 136 societies were identified from previous work [4], 28 from hand searches, and five from other sources [13, 14]. After deduplication, 473 unique societies were identified and subsequently searched for methodological manuals (Fig. 2).

Methodological manuals were available for 98 of these societies and were included in the analysis (Additional file 2). These manuals consisted of 61 handbooks, 29 journal articles, and 8 websites. Most of these documents were relatively recent, with the majority published within the last five years (Table 1).

**Table 1:** General characteristics of included societies and their manuals

**Countries of included societies**	
International societies	29
US	43
UK	11
Canada	6
South Korea	2
Other countries^a^	7
**Specialty of included societies**	
General	9
Oncology	8
Respiratory	7
Cardiology	6
Gastroenterology	6
Radiology	5
Endocrinology	4
Gynaecology /Obstetrics	4
Infectious Diseases	4
Nephrology	3
Neurology	3
Rheumatology	3
Vascular Medicine	3
Critical care	2
Dermatology	2
Haematology	2
Hepatology	2
Nutrition	2
Physical Therapy	2
Preventive Medicine	2
Urology	2
Other fields^b^	17
**Type of manuals**	
Handbook	61
Journal article	29
Website page	8
**Publication year**	
2020–2024	54
2015–2019	21
2010–2014	12
Unclear date	11

^a﻿^Australia, New Zeeland, Brazil, Estonia, Germany, Netherlands, Switzerland

^b^Addiction, Anaesthesia, Cardio-Thoracic Surgery, Emergency, Epidemiology, Family Medicine, Immunology, Occupational Medicine, Orthopaedics, Otorhinolaryngology, Pathology, Paediatrics, Pharmacology, Reproductive, Sleep, Transplantation, Neurosurgery

EO was mentioned in 65 (66%) manuals. The level of detail on the utilization, criteria and terminology varied between them. Some addressed EO in detail, explained the rationale and situations when it can be used for issuing recommendations. On other cases EO was mentioned only briefly. In 30 (31%) documents EO was not mentioned at all, while in 3 (3%) it was unclear whether it is utilized or not.

### Rationale for using EO

Our analysis revealed substantial variation in the utilization of EO in guideline development by societies which mention EO (*n *= 65). The approaches to integrating EO and the rationale for its application demonstrate a considerable heterogeneity across societies. EO is used in various scenarios, when evidence is weak, conflicting, or entirely absent. The utilization of EO can be categorized into two main themes based on their usage rationale:

1. Filling evidence gaps

EO is most commonly employed as a means to address evidence gaps, with 47 (72%) societies using it in this context. These societies utilize EO when reliable data is unavailable, ensuring that actionable guidance can still be provided. For instance:

- American Urological Association (AUA) uses EO as "a statement achieved by panel consensus…for which there is no published evidence."
- The Scottish Intercollegiate Guidelines Network (SIGN) mentions that "if the group feels strongly that they want to make a recommendation even though there is no significant evidence, this should be done as a weak recommendation".
- The National Institute for Health and Care Excellence (NICE) also highlights the use of EO when "there are gaps in the evidence base or subgroups are under-represented."
- The American College of Obstetricians and Gynecologists (ACOG) employs EO in the form of Ungraded Good Practice Statements, which are incorporated “when a practice point is deemed necessary in the case of extremely limited or nonexistent evidence”.
- American Academy of Neurology (AAN) acknowledges that "compelling inferences alone can support practice recommendations without evidence".
- The Centers for Disease Control and Prevention (CDC) develops interim guidelines based on EO stating that “…CDC developed these guidelines using either expert opinion or indirect or emerging evidence, and the recommendations might change when more and better evidence becomes available".
- European Society of Clinical Microbiology and Infectious Diseases (ESCMID) states: "We understand that there might be a clinical need for recommendations even when published evidence is insufficient. In such cases recommendations should be provided with explicit acknowledgment that they are based on expert opinion”.

Similar approaches are used also by many other societies, including the British Society of Allergy and Clinical Immunology (BSACI), American Society of Addiction Medicine (ASAM), the American Heart Association (AHA), British Thoracic Society (BTS), Healthcare Infection Society (HIS) etc. (Additional file 2).

2) Interpretation of evidence

The second theme identified involves the use of EO as a tool for interpreting the available evidence. EO in this context serves to contextualize and synthesize existing evidence, especially when direct conclusions are not easily drawn. This was observed in five societies, with three main examples worth mentioning:

- The Infectious Diseases Society of America (IDSA) highlights that "Expert opinion is not categorized in any of the above classification (i.e., not a level of quality of evidence), but may be critical to interpret studies included in the systematic review".
- U.S. Preventive Services Task Force (USPSTF) mentions “The Task Force is particularly interested in receiving comments on the sufficiency of the systematic review process and interpretation of the body of evidence. However, expert opinion and clinical experience cannot substitute for the body of evidence that the Task Force reviews through a systematic process”.
- The European Reference Network on Rare Endocrine Conditions (Endo-ERN) states that "Expert opinion represents an interpretation of evidence in the context of experts' experiences and knowledge. An expert opinion may be based on the interpretation of studies ranging from uncontrolled case series to randomized controlled clinical trials, thus it is important to describe what type of evidence is being used as the basis for interpretation".

To some extent, a similar approach is followed also by American Physical Therapy Association (APTA) and Society of American Gastrointestinal and Endoscopic Surgeons (SAGES).

For 13 other societies it was not possible to identify how EO is utilized, as no details were provided in their manuals. In such cases EO was mentioned briefly in a sentence. For example, European Society for Medical Oncology (ESMO) mentions only that "Recommendations are based on available scientific data and the authors’ collective expert opinion". Also, The American College of Radiology (ACR) states that "Practice Parameters describe recommended conduct in specific areas of clinical practice. They are based on analysis of current literature, expert opinion, open forum commentary, and informal consensus". In other cases, EO was mentioned only in the system for rating the quality of evidence. For example, in the guidance document of the Royal College of Obstetricians and Gynaecologists (RCOG), EO is mentioned once in the table of “Classification of evidence levels, Level 4: Expert Opinion".

### Foundation of EO recommendations

Regarding how societies come up with the information and the sources which serve as a foundation for EO, most of them rely on more than one distinct foundation, collectively labelled as EO (and other terms, details below). While there is significant variability between societies, there is also a considerable overlap between them, since not only one foundation is used. Due to this, it was not possible to split the societies in groups due to unclear boundaries in this respect. However, based on assessed data, five main foundations could be identified as a potential basis for EO:

1. EO stemming from clinical experience or opinion

This foundation relies on clinical expertise, judgment, and practical experience of guideline panel members. Many of the societies use this foundation (among others). Examples include:

- The American Urological Association (AUA) defines EO as statements based on the clinical training, experience, and judgment of panel members.
- The Heart Rhythm Society (HRS) relies on expert consensus derived from clinical experience and standard of care.
- American Academy of Orthopaedic Surgeons (AAOS) mentions clinical opinion for making statements in such cases.

2) EO stemming from indirect evidence

This foundation incorporates EO when direct (high) quality evidence is unavailable, relying on indirect evidence. For example:

- The European Society of Clinical Microbiology and Infectious Diseases (ESCMID) acknowledges EO when evidence is insufficient, extending empirical evidence to related interventions when appropriate.
- Centers for Disease Control and Prevention (CDC) uses either expert opinion or indirect or emerging evidence for developing interim guidelines.

3) EO stemming from low-quality evidence

This foundation relies on low quality evidence such as small observational studies, case series or case reports etc. For example:

- The World Health Organization (WHO) utilizes EO drawn from case reports and individual or national experiences in the absence of rigorous research evidence.
- The American College of Cardiology (ACC) and American Heart Association (AHA) derive EO from opinion of experts, case studies, and standard of care.
- The Society for Vascular Surgery (SVS) states that committees occasionally have provided guidance using their clinical experience, unsystematic observations, and best interpretation of the low-quality evidence available.
- The American Association of Respiratory Care (AARC) considers indirect evidence from case studies or low-quality data.
- The American Association of Respiratory Care (AARC) use case studies and case reports, acknowledging that recommendation is based on low quality evidence.

4) EO stemming from mechanism-based reasoning or theoretical rationale

This foundation uses principles from physiology, theoretical models, or established scientific rationale to support EO-based recommendations. Although such studies are considered as low-quality evidence, several societies have explicitly presented this as a distinct foundation:

- The American Academy of Neurology (AAN) describes EO as generally accepted principles of care and inferences drawn from known principles of the course of the disease and Bayes’s theorem.
- The Society for Healthcare Epidemiology of America (SHEA) integrates EO based on limited evidence, theoretical rationale, current practices and practical considerations.
- The Society of Interventional Radiology (SIR) includes EO based on physiology, bench research, or first principles.
- The Academy of Nutrition and Dietetics uses EO based usual practice, expert consensus, clinical experience, opinion, or extrapolation from basic research.

5) EO stemming from expert evidence and witnesses

This foundation involves the collection of experience from experts with formulated questions or structured forms, or by involving external experts who provide their experience in a form of a testimony, and using it to inform recommendations when evidence is absent. Examples include:

- The American Society of Hematology (ASH) uses systematically collected expert evidence in areas in which published evidence is insufficient.
- The National Institute for Health and Care Excellence (NICE) incorporates EO from expert witnesses to address gaps in the evidence base.

### Terminology used to describe EO

Among the 65 societies mentioning EO, the terminology used to describe EO varies widely among them (Additional file 3). The most used term is "expert opinion", employed by most societies (*n* = 32, 49%), such as the Centers for Disease Control and Prevention (CDC), European Society of Clinical Microbiology and Infectious Diseases (ESCMID), British Society of Allergy and Clinical Immunology (BSACI), European Society for Radiotherapy and Oncology (ESTRO) etc.

Some societies adopted terms highlighting consensus processes (*n* = 19, 29%), such as for example "consensus" (American Society of Clinical Oncology, ASCO) or "expert consensus" (Society for Vascular Medicine, SVM), or variants like "consensus-based recommendation" (Scottish Intercollegiate Guidelines Network, SIGN) etc.

Others link EO with practice standards, using terms such as "good practice" (British Society for Haematology, BSH) or "best practice" (American Physical Therapy Association, APTA).

Two societies use evidence-based terminology, such as "expert evidence" (American Society of Hematology, ASH) or "expert-based evidence" (European Reference Network on Rare Endocrine Conditions, Endo-ERN). Unique descriptors were also used, such as "inferences from first principles" (American Academy of Neurology, AAN) or "mechanism-based reasoning" (European Association for the Study of the Liver, EASL).

### Evidence grading systems

Given the significant influence of evidence grading systems on how EO is utilized, all evidence grading systems used by the included societies were identified and categorized in this study. Among the 98 methodological manuals, the GRADE system was the most frequently employed, appearing in 52(53%) documents, highlighting its widespread acceptance in guideline development. In contrast, 6 (6%) societies utilized their own unique systems, while 12 (12%) societies employ modified versions of established systems, including GRADE, AHA, OCEBM, IDSA and ESC. This could reflect a preference among some societies for tailoring systems to meet specific needs. Other grading systems are less commonly used, such as ACC/AHA, SIGN, OCEBM, USPSTF, ASTRO, GLIDES, IDSA/USPHSG, and ICSIG. A small number of societies utilize multiple systems within the same guidance document, while 7 documents (8%) have no information on the system.

**Fig. 3:**
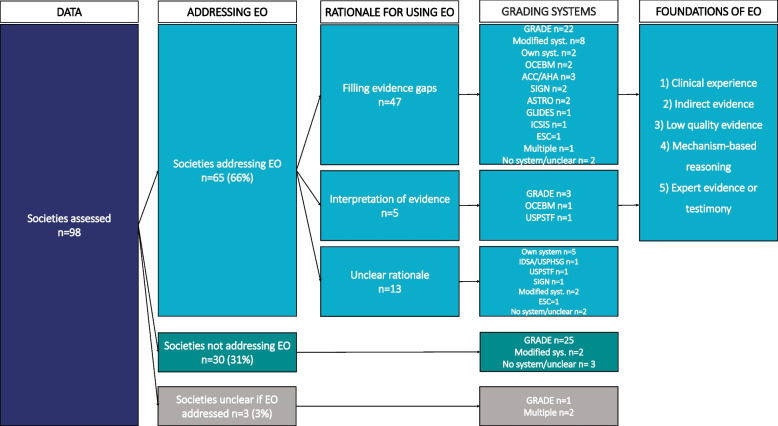
Visual description of EO utilization and evidence grading systems. ACC: American College of Cardiology, AHA: American Heart Association, ASTRO: American Society for Radiation Oncology, AUA: American Urological Association, EO: Expert Opinion, GLIDES: Guidelines Into Decision Support, GRADE: Grading of Recommendations, Assessment, Development, and Evaluations, ICSIS: Institute for Clinical Systems Improvement System grading approach, IDSA-USPHS: The Infectious Diseases Society of America-US Public Health Service, OCEBM: Oxford Centre for Evidence-Based Medicine, SIGN: Scottish Intercollegiate Guidelines Network, USPSTF: United States Preventive Services Task Force

Further analysis revealed some differences between societies which use EO to fill evidence gaps and those that do not mention EO (Fig. 3). Among the 47 societies in the first group, GRADE was the most prevalent, being employed by 22 societies (47%), followed by 8 societies using various modifications of current systems. The rest of societies in this group use other systems. On the other hand, for societies that do not address EO in their manuals, evidence grading systems were less varied, and GRADE was used in 25 (83%) societies (Fig. 3).

### Proposed definition of EO

Based on the findings of this study, a definition of the EO concept emerged on how EO is currently conceptualized and utilized by national and international societies. Although societies use various terms for EO, we propose this definition for EO as a concept: *"The concept of expert opinion in clinical guidelines refers to the synthesis of guidance derived from clinical experience, expert judgment, indirect evidence, very low-quality evidence or mechanism-based reasoning, aimed at supporting clinical decision-making in the absence of evidence or when inconclusive evidence requires contextualization and interpretation*”.

## Discussion

### Summary of findings

In this study, we systematically searched 473 unique national and international societies involved in guideline development and identified 98 methodological manuals. We assessed these manuals to examine the rationale and foundations of EO. Our findings reveal that EO is mentioned or utilized by 66% of the included societies. Among these, two-thirds use EO for filling evidence gaps. There is considerable variability in how societies integrate EO into their methodological processes. We identified five primary foundations of EO—clinical experience, indirect or low-quality evidence, mechanism-based reasoning, and systematically collected expert evidence/testimony—highlighting the heterogeneity in its conceptualization. Finally, our analysis led to the development of a comprehensive and novel definition of EO, capturing its role and the foundations upon which it is built.

### Comparison with other studies

Although no other study has used this approach to assess EO application in clinical guidelines, several authors have addressed this topic. For example, Schünemann et al. distinguish EO from evidence and define EO solely as opinion, describing it as “a view or judgment formed about something, not necessarily based on facts”. They also propose that when no evidence is available, panel members' experience should be collected in structured forms, which they classify as expert evidence rather than EO, to issue recommendations [6]. Our findings, however, indicate that EO is not merely opinion (even if the name suggest as such), as it encompasses multiple sources, including low-quality or inconclusive evidence. This perspective is reflected in our proposed definition, which considers EO a broader concept rather than being restricted to personal judgment. While we agree with Schünemann et al. that structured collection of experience enhances methodological rigor, we still consider expert evidence a form of EO rather than a separate entity.

On the other hand, Djulbegovic et al. argue against distinguishing between evidence-based and consensus-based guidelines, asserting that evidence never speaks for itself and always requires interpretation—particularly when the balance between benefits and harms is close [15]. While we agree with this principle and acknowledge it in our definition, their argument does not fully capture the scope of EO. Our findings show that EO is frequently used not just for interpreting evidence but also for issuing recommendations in the complete absence of evidence. This highlights EO’s much broader role in guideline development.

Robert M. Levy presents another relevant viewpoint, contending that EO is not inherently a substandard tool for medical guidelines and that panel members typically consider all available literature alongside their experience [16]. We agree that EO plays a crucial role in guideline development and that guideline panels integrate available evidence. However, our results reveal significant variability in EO application, indicating a lack of standardization. It is this inconsistency, rather than EO itself, that makes it a suboptimal tool in its current state. For EO to become a more reliable component of guideline development, standardization is essential. Until such standardization is achieved, this issue will remain controversial.

### Implications

Our findings challenge the notion that EO should be entirely excluded from guideline development, as it remains a significant component across most societies. The enduring presence of EO in guideline development and its widespread use to fill evidence gaps (even by societies which use frameworks such as GRADE which does not accommodate EO explicitly) highlight its practical necessity. This suggests that an outright dismissal of EO is neither realistic nor transparent.

At the same time, our study reveals substantial variation in how societies define and apply EO, reflecting ambiguities and inconsistencies in its role within guideline development. Many societies fail to provide clear criteria for its use, leading to possible inconsistencies both within and between guidelines. This risks undermining the reliability, transparency, and methodological rigor of guideline development.

These findings underline a pressing need to bridge this divide between these two schools of thought which currently dominate, one that rejects EO entirely and another that embraces it without defined criteria. This necessitates a re-evaluation and redefinition of the role of EO in guideline development, as a first step towards standardization, to ensure that EO, when used, is applied transparently and consistently. This would not only accommodate the practical needs of guideline-developing societies but also enhance the integrity and applicability of their clinical guidelines.

Future frameworks for EO standardization could build upon the definition proposed in this study, which emerged from a systematic assessment of societal practices. This definition can serve as a foundation for developing clearer methodological guidance, addressing a critical gap in guideline development.

### Strengths and limitations

To our knowledge, this is the first study to systematically examine EO in methodological manuals and propose a data-driven definition, laying the groundwork for future frameworks. Another key strength of this study is the systematic approach with multiple steps to identify manuals from different sources. This approach made it possible to create the most comprehensive map of methodological manuals to date, spanning across various medical disciplines and geographic regions of the World. This provides a unique opportunity for other researchers to sample data.

However, several limitations must be acknowledged. First, the study was limited to manuals available in English, which may restrict its generalizability to societies that use other languages. Second, 33 societies did not mention or specify the use of EO in their manuals. Whether some of these societies might utilize EO when developing their guidelines remains unknown. This could have potentially underestimated our findings, as EO could have a larger role than shown in this study. Third, although our literature searches were extensive, incorporating multiple layers and diverse sources, some societies may have been missed, and certain methodological manuals may have remained unidentified due to the varied ways societies publish these documents.

## Conclusions

This study highlights the essential role of EO in clinical guideline development while revealing inconsistencies in its application. The widespread use of EO to fill evidence gaps and interpret ambiguous evidence underscores its necessity. However, the variability in how EO is applied across societies, coupled with the absence of criteria, risks undermining guideline quality and consistency. To address these challenges, we propose a comprehensive EO definition as a first step toward standardization, aiming to enhance guideline consistency, transparency, and clinical decision-making.

## Supplementary Information

Additional file 1. Protocol.

Additional file 2. Included societies and details on guidance manuals and utilization of EO.

Additional file 3. EO terminology.

## Data Availability

The data for this study was obtained from methodological manuals which are freely available and accessible. The complete list of included manuals alongside their URLs, DOIs and main extracted information is provided in the additional files.

## Abbreviations

ACC: American College of Cardiology
AHA: American Heart Association
ASTRO: American Society for Radiation Oncology
AUA: American Urological Association
EO: Expert Opinion
GLIDES: Guidelines Into Decision Support
GRADE: Grading of Recommendations, Assessment, Development, and Evaluations
ICSIS: Institute for Clinical Systems Improvement System grading approach
IDSA-USPHS: The Infectious Diseases Society of America-US Public Health Service
OCEBM: Oxford Centre for Evidence-Based Medicine
SIGN: Scottish Intercollegiate Guidelines Network
USPSTF: United States Preventive Services Task Force
