# Supplementary material for: Defining expert opinion in clinical guidelines: insights from 98 scientific societies – a methodological study
Source: BMC Med Res Methodol. 2025 Apr 2;25:87. doi: 10.1186/s12874-025-02534-0 (PMC11963610; doi:10.1186/s12874-025-02534-0)
Supplement: Supplementary file 1 — Additional file 1. Protocol. [file 12874_2025_2534_MOESM1_ESM.docx]

**Additional file 1 - Protocol**

## Aim

This Work Package (WP) will provide an exhaustive overview of existing directives governing the utilisation of EO within clinical guidelines, as stipulated by a variety of societies and organisations. To fulfil this, a systematic approach will be undertaken, encompassing systematic literature searches designed to identify relevant guidance documents (e.g. manuals, handbooks) on guideline methodology regarding utilisation of EO, published by societies and organisations involved in guideline development.

## Identification of guidance documents on guideline development methodology

The focus of this WP is assessment of guidance documents of various societies and organisations which develop guidelines. To systematically search for such documents, a list of societies and organisations involved in guideline development will be compiled. This will be done in three steps:

**Step 1: Searching and screening of clinical guidelines**

PubMed will be searched for guidelines in all topics published in the last 5 years (2019-2024), using a broad search string.

Search Strategy:

PubMed search

Date: 12.03.2024

| Search | Query | Results |
| --- | --- | --- |
| #1 | Search: (guideline[Title]) Filters: Guideline, from 2019 - 2024  Sort by: Publication Date | [**965**](https://pubmed.ncbi.nlm.nih.gov/?term=%28guideline%5BTitle%5D%29&filter=pubt.guideline&filter=years.2019-2024&sort=pubdate&size=200) |

Screening:

The retrieved references (guidelines) will be screened for relevancy in title/abstract and full text by one reviewer, using Rayyan (criteria below):

Inclusion criteria:

- Clinical Guidelines (exclude: HTAs, articles, etc).
- Published by a society or organisation (national or international)
- Any topic in internal medicine or surgery (exclude: infection prevention, dentistry, psychology, psychiatry, nutrition).
- Any country or region in the World.
- Published from 2019 onwards.
- Published in English (or abstract in English).
- If multiple updates, the latest version will be used.

**Step 2: Identification of societies**

All relevant clinical guidelines (fulfilling the criteria) will be listed, and the names of societies and organisations mentioned in these documents will be extracted and listed. Known societies (from previous work) will also be added to the list (and deduplicated).

**Step 3 Searching guidance documents**

All national or international societies/organisations, identified from the published guidelines, will be checked if they have any guidance published on EO utilisation. Two reviewers will check independently.

For each society/organisation we will:

- Identify and search the respective society website for guidance documents on guideline methodology.
- Google will be searched for guidance documents published by the society.

Search method:

1) Check society website. If website in a foreign language use Google Translate. If it can’t be translated exclude (see exclusion reasons).

2) Check source the methods section of the source guideline.

3) Search Google Advanced (string below):

All these words: society's name

Any of these words: manual, methods, methodology, guidance, procedure development developing guidelines sop procedure.

Inclusion criteria:

- Any guidance document, manual, handbook or article regarding the methodology by which the society develops clinical guidelines.
- Published in English.
- If multiple versions the latest one will be used.

## Data extraction

Data extraction will be carried out by a designated reviewer, in piloted forms in Excel, and checked by another.

The following information will be extracted for each identified guidance document (non-exhaustive list):

- Organisations/Society
- Source (PubMed, website)
- URL
- Year of publication
- Document type (manual, handbook, other)
- System of grading evidence
- EO allowed
- EO part of grading system
- EO definition
- Criteria when EO can be used (if yes, how)
- Narrative description of EO utilisation

## Data analysis

The identified guidance documents will be thoroughly read and analysed if and how they regulate EO utilisation. The analysis and presentation of data will be conducted through narrative evidence synthesis methodologies.
