# Supplementary material for: Defining expert opinion in clinical guidelines: insights from 98 scientific societies – a methodological study
Source: BMC Med Res Methodol. 2025 Apr 2;25:87. doi: 10.1186/s12874-025-02534-0 (PMC11963610; doi:10.1186/s12874-025-02534-0)
Supplement: Supplementary file 2 — Additional file 2. Included societies and details on guidance manuals and utilization of EO. [file 12874_2025_2534_MOESM2_ESM.docx]

**Additional file 2 - Included societies and details on guidance manuals and utilization of EO (n=98)**

| **No.** | **Society** | **Country based** | **Document title** | **URL/DOI** | **Publication date** | **EO mentioned** | **EO name** | **EO utilization (quote)** |
| --- | --- | --- | --- | --- | --- | --- | --- | --- |
| 1 | American College of Cardiology (ACC) | US | Methodology Manual and Policies From the ACCF/AHA Task Force on Practice Guidelines | <https://professional.heart.org/-/media/PHD-Files/Guidelines-and-Statements/methodology_manual_and_policies_ucm_319826.pdf> | 2010 | yes | Expert opinion | "Consensus opinion of experts, case studies, or standard of care" |
| 2 | American Heart Association (AHA) | US | Methodology Manual and Policies From the ACCF/AHA Task Force on Practice Guidelines | <https://professional.heart.org/-/media/PHD-Files/Guidelines-and-Statements/methodology_manual_and_policies_ucm_319826.pdf> | 2010 | yes | Expert opinion | "Consensus opinion of experts, case studies, or standard of care" |
| 3 | Society for Vascular Surgery (SVS) | US | Guideline methodology of the Society for Vascular Surgery including the experience with the GRADE framework | <https://www.jvascsurg.org/article/S0741-5214(11)00192-3/fulltext> | 2011 | yes | Consensus statement | "When direct comparative evidence is not available to guide clinical recommendations, committees occasionally have provided guidance using their clinical experience, unsystematic observations, and best interpretation of the low-quality evidence available in a particular surgical area". |
| 4 | Congress of Neurological Surgeons (CNS) | US | Guideline Development Methodology Endorsed by the American Association of Neurological Surgeons (AANS), the Congress of Neurological Surgeons (CNS), and the AANS/CNS Joint Guideline Committee | <https://www.cns.org/Assets/7877b6bf-1ec3-45de-b524-6543ce7fd6df/636982811309170000/jgcguidelinedevelopmentmethodology-pdf> | 2012 | yes | Expert opinion | "Level III (or C) Recommendation- Evidence from case series, comparative studies with historical controls, case reports, and expert opinion, as well as significantly flawed randomized controlled trials". |
| 5 | American Academy of Otolaryngology - Head and Neck Surgery (AAO-HNS) | US | Clinical Practice Guideline Development Manual, Third Edition: A Quality-Driven Approach for Translating Evidence into Action | <https://doi.org/10.1177/0194599812467004> | 2013 | yes | Expert opinion | "Using expert opinion or consensus to fill evidence gaps is entirely appropriate, provided this basis is explicit and transparent to the critical reader". |
| 6 | International Late Effects of Childhood Cancer Guideline Harmonization Group (IGHG) | Unclear | A Worldwide Collaboration To Harmonize Guidelines For The Long-Term Follow-Up Of Childhood And Young Adult Cancer Survivors: A Report From The International Late Effects Of Childhood Cancer Guideline Harmonization Group | <https://doi.org/10.1002/pbc.24445> | 2013 | yes | Expert opinion | Table 1 - Criteria for grading the levels of evidence and strength of recommendations: "GRADE C Evidence from studies with serious flaws. Only expert opinion, or standards of care". "Strong recommendation based on expert opinion". |
| 7 | American College of Chest Physicians (CHEST) | US | Methodologies for the Development of CHEST Guidelines and Expert Panel Reports | <https://doi.org/10.1378/chest.14-0824> | 2014 | yes | Consensus | "Many topic areas have no or weak supporting evidence; however, there is often need for credible guidance regarding clinical questions when there is insufficient evidence to support a formal guideline". "GOC also recognized the need for a hybrid approach combining evidencebased CPG methodologies and consensus-based methods when evidence is variable across subtopics within a larger project scope". |
| 8 | World Health Organization (WHO) | Worldwide | WHO Handbook for Guideline Development | <https://www.who.int/publications/i/item/9789241548960> | 2014 | yes | Expert opinion/Good practice statements | 1) Emergency guidelines: "...or may need to issue recommendations based on expert opinion only. 2) Regular guidelines: "When no evidence is available, this should be made clear and the basis for any recommendations made should be presented. For example, in the absence of rigorous research evidence, case reports and a compilation of national or individual experiences may be the basis for formulating a (conditional) recommendation". |
| 9 | European Society for Clinical Nutrition and Metabolism (ESPEN) | Luxemburg | Standard operating procedures for ESPEN guidelines and consensus papers | <https://www.espen.org/files/ESPEN-Guidelines/0__Standard_operating_procedures_for_ESPEN_guidelines_and_consensus_papers_2.pdf> | 2015 | yes | Expert opinion | "Good Practice Points: Recommended best practice based on the clinial experience of the guideline development group". |
| 10 | American Urological Association (AUA) | US | American Urological Association Clinical Practice Guidelines Development | <https://www.auanet.org/documents/education/guidelines-SOP.pdf> | 2015 | yes | Expert opinion | "A statement achieved by panel consensus that is based on members' clinical training, experience, knowledge and judgment and for which there is no published evidence". |
| 11 | American College of Radiology (ACR) | US | Practice Parameters and Technical Standards Handbook | <https://www.acr.org/-/media/ACR/Files/Practice-Parameters/DevelopmentHandbook.pdf> | 2015 | yes | Expert opinion | "Practice Parameters describe recommended conduct in specific areas of clinical practice. They are based on analysis of current literature, expert opinion, open forum commentary, and informal consensus". |
| 12 | European Society of Endocrinology (ESE) | UK | ESE guidelines, why and how | <https://doi.org/10.1530/EJE-15-0625> | 2015 | yes | Good Practice Statement | "However, it is important to emphasize a lack of solid evidence in the guideline, to acknowledge the larger role of expert opinions and to resist strong recommendations in these cases. As such, it is acknowledged that a good practice statement should go without formal assessment of the evidence, as the lack of apparent evidence might wrongly suggest the lack of a good rationale for clinical practice." |
| 13 | European Association for Cardio-Thoracic Surgery (EACTS) | UK | Methodology manual for European Association for Cardio-Thoracic Surgery (EACTS) clinical guidelines | <https://doi.org/10.1093/ejcts/ezv309> | 2015 | yes | Consensus of expert opinion | "Table 3: Levels of evidence. Level of evidence C: Consensus of expert opinion, and/or small studies, retrospective studies, registries". "Expert consensus statements describe an expert position on an issue where there is controversy/uncertainty and where high-level evidence is not available to guide clinical recommendations". |
| 14 | Caring for Australasians with Renal Impairment | Australia and New Zeeland | KHA-CARI Guidelines Development Manual | <https://www.cariguidelines.org/wp-content/uploads/2020/06/KHACARI_Guideline_development_-manual.pdf> | 2015 | yes | Ungraded Suggestions for Clinical Care | "In the KHA-CARI guideline, an ungraded statement is separated from the graded recommendations and suggestions in a section titled “UNGRADED SUGGESTIONS FOR CLINICAL CARE”. This ensures that there is a clear distinction between evidence-based and opinion based statements". |
| 15 | Academy of Nutrition and Dietetics | US | Academy of Nutrition and Dietetics Methodology for Conducting Systematic Reviews for the Evidence Analysis Library | <https://doi.org/10.1016/j.jand.2015.11.008> | 2016 | yes | Expert opinion | "Conclusion based on usual practice, expert consensus, clinical experience, opinion, or extrapolation from basic research". |
| 16 | Korean Society of Radiology | South Korea | Methodology for Developing Evidence-Based Clinical Imaging Guidelines: Joint Recommendations by Korean Society of Radiology and National Evidence- Based Healthcare Collaborating Agency | <https://doi.org/10.3348/kjr.2017.18.1.208> | 2017 | yes | Expert opinion | "Table 4. Criteria for Evidence Level of Each Evidence Literatures - Level 5: Expert Opinion". |
| 17 | National Evidence-based Healthcare Collaborating Agency (NECA) | South Korea | Methodology for Developing Evidence-Based Clinical Imaging Guidelines: Joint Recommendations by Korean Society of Radiology and National Evidence- Based Healthcare Collaborating Agency | <https://doi.org/10.3348/kjr.2017.18.1.208> | 2017 | yes | Expert opinion | "Table 4. Criteria for Evidence Level of Each Evidence Literatures - Level 5: Expert Opinion". |
| 18 | American Academy of Neurology (AAN) | US | 2017 Edition Clinical Practice Guideline Process Manual | <https://www.aan.com/link/106dc264682244459ed413bcbed1c39f.aspx> | 2017 | yes | Inferences from first principles | "There are unusual circumstances where compelling inferences alone can support practice recommendations without evidence". Definition: "Generally accepted principles of care. These are labeled PRIN in the rationale section". "These inferences are not derived from evidence as defined in the EBM context; instead, they are inferred from known principles of the course of the disease and Bayes’s theorem (an important principle regarding contingent probabilities)." |
| 19 | Society of Interventional Radiology (SIR) | US | Standards 2.0: Methodology Update | <https://doi.org/10.1016/j.jvir.2018.05.018> | 2018 | yes | Expert opinion | "Expert consensus based on clinical practice". "Expert opinion without explicit critical appraisal or based on physiology, bench research, or first principles". |
| 20 | American Society for Radiation Oncology (ASTRO) | US | ASTRO Clinical Practice Guideline Methodology Guide | <https://www.astro.org/ASTRO/media/ASTRO/Patient%20Care%20and%20Research/PDFs/ASTRO_GuidelineMethodology.pdf> | 2019 | yes | Expert opinion | "Expert opinion recommendations are used where guidance is considered essential due to factors such as high prevalence, mortality, or morbidity but either relevant data do not exist, the available evidence does not reflect current technology/practice, or there is substantial variation in practice or controversy". |
| 21 | Scottish Intercollegiate Guidelines Network (SIGN) | UK | SIGN50 A guideline developer’s handbook | <https://www.sign.ac.uk/media/2038/sign50_2019.pdf> | 2019 | yes | Consensus based recommendation | "If the group feels strongly that they want to make a recommendation even though there is no significant evidence, this should be done as a weak recommendation"."Note that there must be some evidence of opinion supporting the recommendation from outside the guideline group. If no such evidence exists, formal methods should be used to develop a consensus-based recommendation which will be clearly identified as such within the guideline by a statement accompanying the recommendation" |
| 22 | European Society of Regional Anaesthesia and Pain Therapy (ESRA) | Switzerland | Development of evidence-based recommendations for procedure-specific pain management: PROSPECT methodology | <https://doi.org/10.1111/anae.14776> | 2019 | yes | Clinical Practice Information | "Table 1 Relationship between quality of the study and levels of evidence and grades of recommendation-Clinical practice information (expert opinion), inconsistent evidence". |
| 23 | European Association for the Study of the Liver (EASL) | Switzerland | Clinical Practice Guidelines of the European Association for the study of the Liver – Advancing methodology but preserving practicability | <https://doi.org/10.1016/j.jhep.2018.10.011> | 2019 | yes | Expert opinion | "These documents aim to help clinicians to optimise their management of patients with liver diseases, based on the most current scientific evidence and, where evidence is scarce, consensus opinion of experts from the field". |
| 24 | American Society of Hematology (ASH) | US | Methodology for the American Society of Hematology VTE guidelines: current best practice, innovations, and experiences | <https://doi.org/10.1182/bloodadvances.2020001768> | 2020 | yes | Expert evidence | "Each guideline recommendation was supported by a systematic review of the effects of interventions, including use of indirect evidence or systematically collected expert evidence in areas in which published evidence was insufficient". |
| 25 | Royal College of Obstetricians and Gynaecologists (RCOG) | UK | Developing a Green-top Guideline - Guidance for developers | <https://www.rcog.org.uk/media/4xyn1qmb/rcog-guideline-development-guide.pdf> | 2020 | yes | Expert opinion | Appendix 7 Classification of evidence levels: "Level 4 - Expert Opinion" |
| 26 | European Reference Network on Rare Endocrine Conditions (Endo-ERN) | The Netherlands | Handbook #4: Methodology for the Development of Clinical Practice Guidelines for Rare or Low-Prevalence and Complex Diseases | <https://health.ec.europa.eu/document/download/9d7de9a7-de0d-49b5-a297-5a0303e3059b_en?filename=ern_cpg-cdst_hb4_en.pdf> | 2020 | yes | Expert-based evidence | "Expert opinion represents an interpretation of evidence in the context of experts' experiences and knowledge. An expert opinion may be based on the interpretation of studies ranging from uncontrolled case series to randomized controlled clinical trials, thus it is important to describe what type of evidence is being used as the basis for interpretation". |
| 27 | International Society for Heart and Lung Transplantation (ISHLT) | US | International Society for Heart and Lung Transplantation Policies and Processes for Development and Endorsement of Professional Practice Guidelines and Consensus Statements | <https://www.ishlt.org/docs/default-source/uploadedfiles/documents/s-g-document-development-policies-and-procedures.pdf?sfvrsn=283504b7_0#0> | 2020 | yes | Expert opinion | "A comprehensive literature search and expert opinion which provides the evidence for graded recommendations". |
| 28 | Healthcare Infection Society (HIS) | UK | Healthcare Infection Society Guideline Development Manual (V14) | <https://his.org.uk/media/mr4jph0r/his_guideline-methodology_v-14.pdf> | 2020 | yes | Expert opinion | "Formal consensus outside the group using Delphi technique or the nominal-group technique: this involves seeking to explore the views of the stakeholders outside of the GDG group with the decision agreed by the Guideline Committee who in such circumstances provide the quality assurance for the guideline. This method is generally used when the literature searches identified no evidence and the recommendation is based on an expert opinion". "Good Practice Points (GPP): to assist guideline users by providing short pieces of advice which may not have an evidence base, but which are considered essential to good clinical practice". |
| 29 | Heart Rhythm Society (HRS) | US | HRS Clinical Document Development Methodology Manual and Policies | <https://www.hrsonline.org/sites/default/files/2021-05/HRS%20Clinical%20Document%20Methodology%20Manual_March%202021_0.pdf> | 2021 | yes | Expert opinion | "Consensus of expert opinion based on clinical experience, standard of care, or when evidence is insufficient, vague, or conflicting". |
| 30 | Society for Cardiovascular Magnetic Resonance (SCMR) | US | Standards for writing Society for Cardiovascular Magnetic Resonance (SCMR) endorsed guidelines, expert consensus, and recommendations: a report of the publications committee | <https://doi.org/10.1186/s12968-021-00801-9> | 2021 | yes | Expert consensus statement | "Expert consensus statements are meant to fill in critical practice gaps when there is insufficient published data to provide the recommendations with the level of evidence expected from guidelines. These recommendations are often based on a combination of systematic review of the available literature, as well as a comprehensive synthesis of existing research, accepted best practice patterns, as well as expert opinion". |
| 31 | The American College of Obstetricians and Gynecologists (ACOG) | US | Clinical Practice Guideline Methodology | <https://doi.org/10.1097/AOG.0000000000004519> | 2021 | yes | Ungraded Good Practice Statements | "Ungraded Good Practice Statements also may be incorporated when a practice point is deemed necessary in the case of extremely limited or nonexistent evidence". |
| 32 | Infectious Diseases Society of America (IDSA) | US | IDSA Handbook for Clinical Practice Guidelines Development | <https://idsociety.sharepoint.com/:b:/s/CAPG/EaXC004vbMdPna7PsrvQ34EBhl2yd5WOtyeUrEOK2rE2IQ?e=b9x6lT> | 2021 | yes | Expert opinion | "Expert opinion is not categorized in any of the above classification (i.e., not a level of quality of evidence), but may be critical to interpret studies included in the systematic review". |
| 33 | European Society of Gastrointestinal and Abdominal Radiology (ESGAR) | Austria | ESGAR guidelines for development of consensus guidelines | <https://esgar.org/fileadmin/media/Research/2021_Guidelines_for_development_of_consensus_guidelines.pdf> | 2021 | yes | Expert opinion | "When the available literature is deemed to be limited and/or of low quality, the committee may base statements on consensus opinion (derived from the completed questionnaires) even if these contradict the available low quality evidence, and justify this in explanatory text". |
| 34 | British Society of Allergy and Clinical Immunology (BSACI) | UK | British Society for Allergy and Clinical Immunology (BSACI) Standards of Care Committee (SOCC) Guideline Production Manual | <https://www.bsaci.org/wp-content/uploads/2021/04/BSACI-guideline-production-manual-2021-version-1.2-MS.pdf> | 2021 | yes | Expert opinion | "Recommended best practice based on the clinical experience of the guideline development group". |
| 35 | U.S. Preventive Services Task Force (USPSTF) | US | U.S. Preventive Services Task Force Procedure Manual | <https://www.uspreventiveservicestaskforce.org/uspstf/procedure-manual> | 2021 | yes | Opinions of respected authorities | "The Task Force invites and considers the opinions of the public and experts throughout the recommendation development process, including the draft evidence review and the draft recommendation statement. The Task Force is particularly interested in receiving comments on the sufficiency of the systematic review process and interpretation of the body of evidence. However, expert opinion and clinical experience cannot substitute for the body of evidence that the Task Force reviews through a systematic process". |
| 36 | British Thoracic Society (BTS) | UK | British Thoracic Society Standards of Care Committee Guideline Production Manual | <https://www.brit-thoracic.org.uk/media/455938/bts-guideline-production-manual-2022.pdf> | 2022 | yes | Consensus | "Where there is a lack of evidence on a particular outcome, the GDG should be clear about how a consensus has been reached in formulating a recommendation (for example using the Delphi process)". "If the GDG feels strongly that they want to make a recommendation even though there is no significant evidence, this should be presented as a ‘Conditional’ recommendation (please see Item 4.8.2) and marked as ‘Conditional – by consensus’. There should be some evidence of opinion supporting the recommendation from outside the GDG. If no such evidence exists, formal methods should be used to develop a consensus-based recommendation and these methods will be clearly identified as such within the Guideline" |
| 37 | Centers for Disease Control and Prevention (CDC) | US | Standards Required for the Development of CDC Evidence-Based Guidelines | <https://doi.org/10.15585/mmwr.su7101a1> | 2022 | yes | Expert opinion | "The term interim implies that CDC developed these guidelines using either expert opinion or indirect or emerging evidence, and the recommendations might change when more and better evidence becomes available". |
| 38 | Society for Healthcare Epidemiology of America (SHEA) | US | Handbook for SHEA-Sponsored Expert Guidance, Consensus, and Guideline Documents | <https://shea-online.org/wp-content/uploads/2024/11/2024-Handbook-Final.pdf> | 2022 | yes | Expert Guidance | "Expert Guidance provide practice recommendations in the absence of availability of literature to support a formal guideline...". "Expert Guidance is based on a synthesis of limited evidence, theoretical rationale, current practices, practical considerations, writing group opinion, and consideration of potential harm". |
| 39 | European Association of Urology (EAU) | The Netherlands | European Association of Urology Guidelines Office Development Handbook | <https://d56bochluxqnz.cloudfront.net/media/Guidelines_Office_Development_Handbook_website.pdf> | 2022 | yes | Expert opinion or consensus | "Expert opinion or consensus finding outcomes may be used to make recommendations in topics with gaps in the evidence, however the strength of the recommendation will be limited. Discussing topics with limited evidence allows guideline developers to highlight future research needs and suggest how to best fill existing gaps. The guideline as a whole, however, must avoid over-reliance on expert opinion or clinical consensus as a primary decision making strategy" |
| 40 | American Society of Clinical Oncology (ASCO) | US | ASCO Guidelines Methodology Manual | <https://society.asco.org/sites/new-www.asco.org/files/content-files/practice-and-guidelines/documents/2023-Guidelines-Methodology-Manual-4-5-23.pdf> | 2022 | yes | Consensus | "In clinically important areas where there is limited evidence or a lack of high-quality evidence to inform clinical guidance recommendations, ASCO uses a formal consensus methodology based on the modified Delphi technique". "If the quality of the evidence identified through the systematic review is insufficient to inform recommendations, a consensus methodology may be utilized". |
| 41 | Society for Integrative Oncology (SIO) | US | Society for Integrative Oncology (SIO) Guidelines Methodology Manual | <https://integrativeonc.org/wp-content/uploads/2023/09/sio-guidelines-methodology-manual-final-v1.pdf> | 2022 | yes | Consensus opinion of experts | "Consensus opinion of experts may be reasonable to provide guidance on the topic until better evidence is available". |
| 42 | Kidney Disease Improving Global Outcomes (KDIGO) | Belgium | KDIGO Methods Manual For Guideline Development | <https://kdigo.org/wp-content/uploads/2022/12/KDIGO-Methods-Manual-for-Guideline-Development_v1-3-copy-1.pdf> | 2022 | yes | Expert consensus opinion | "For intervention reviews, where it is likely that there will be insufficient RCTs to inform decision making, the WG should consider what level of evidence (in terms of study design) they will consider appropriate to include (and will enable a recommendation to be made) before relying on expert consensus opinion". |
| 43 | Cancer Care Alberta | Canada | Guideline Methodology Handbook | <https://www.albertahealthservices.ca/assets/info/hp/cancer/if-hp-cancer-guide-utilization-handbook.pdf> | 2022 | yes | Expert opinion | "The Working Group members formulate the guideline recommendations based on existing published guidelines and the evidence synthesized by the KMS blended with expert clinical experience and local context". |
| 44 | UK Kidney Association | UK | Clinical Practice Guideline Development Manual | <https://ukkidney.org/sites/renal.org/files/UK%20Kidney%20Association%20Guideline%20Development%20Manual%20October%202021.pdf> | 2022 | yes | Expert opinion | "Grade D evidence is based only on case studies or expert opinion". |
| 45 | European Society for Vascular Surgery (ESVS) | France | Editor's Choice – European Society for Vascular Surgery Clinical Practice Guideline Development Scheme: An Overview of Evidence Quality Assessment Methods, Evidence to Decision Frameworks, and Reporting Standards in Guideline Development | <https://doi.org/10.1016/j.ejvs.2022.03.014> | 2022 | yes | Consensus of experts | "In the event of weak, or even lack of, evidence on a clinical question that is nevertheless considered clinically important, it is possible to issue a recommendation based solely on the expert opinion". |
| 46 | European Society of Cardiology (ESC) | France | ESC Clinical Practice Guidelines: Policies and Procedures | <https://www.escardio.org/Guidelines/Clinical-Practice-Guidelines/Guidelines-development/Writing-ESC-Guidelines> | 2022 | yes | Consensus of opinion | "ESC guideline reference table 2 - Level of Evidence C: Consensus of opinion of the experts and/or small studies, retrospective studies, registries". |
| 47 | American College of Occupational and Environmental Medicine | US | Methodology | <https://acoem.org/Practice-Resources/Practice-Guidelines-Center/Guidelines-Methodology> | 2022 | yes | Consensus recommendation | "When the disorder with quality evidence is similar, but not identical to another disorder without quality evidence, "I-rated guidance" (i.e., expert consensus) may be developed based on expert opinion". |
| 48 | American Academy of Orthopaedic Surgeons (AAOS) | US | AAOS Clinical Practice Guideline Methodology | <https://www.aaos.org/globalassets/quality-and-practice-resources/methodology/cpg-methodology-september-2023.pdf> | 2023 | yes | Consensus | "In the absence of sufficient evidence, the guideline work group is making a statement based on their clinical opinion". |
| 49 | American College of Rheumatology (ACR) | US | American College of Rheumatology Policy and Procedure Manual for Clinical Practice Guidelines | <https://assets.contentstack.io/v3/assets/bltee37abb6b278ab2c/bltae11ca9142708dfa/clinical-practice-guideline-policy-procedure-manual.pdf> | 2023 | yes | Expert opinion | "When there is no high-quality evidence and expert opinion is more heavily required when formulating a recommendation, this will be transparently presented both in the guideline development process". |
| 50 | European Society for Medical Oncology (ESMO) | Switzerland | ESMO Guidelines Committee (GLC) Standard Operating Procedures (SOPs) for ESMO Clinical Practice Guidelines (CPGs) | <https://www.esmo.org/content/download/77789/1426712/file/ESMO-Clinical-Practice-Guidelines-Standard-Operating-Procedures.pdf> | 2023 | yes | Expert opinion | "Recommendations are based on available scientific data and the authors’ collective expert opinion". |
| 51 | American Association of Respiratory Care (AARC) | US | A Process Manual for Clinical Practice Guidelines Development | <https://www.aarc.org/wp-content/uploads/2023/01/aarc-clinical-practice-guidelines-development.pdf> | 2023 | yes | Opinion | "When little evidence is found, options moving forward are to state that further research is needed, search for lower quality evidence via case studies and case reports, acknowledging that poor evidence was found upon which the recommendation is based, or expanding the population or intervention in the search with intending to use indirect evidence (opinions) as the basis of judgments". |
| 52 | American Physical Therapy Association (APTA) | US | APTA Clinical Practice Guideline Process Manual | <https://www.apta.org/contentassets/ab3a561c2a5e4cb5928d2bd2da93ab7d/apta-cpg-manual-2022.pdf> | 2023 | yes | Best practice | "Exceptional situations where validating studies have not or cannot be performed and there is a clear benefit, harm, or cost; or when expert opinion is needed to support the clinical decision-making process. This could include situations of conflicting evidence or when the GDG wants to specify conditions under which select actions may be taken". "Theoretical / foundational: A preponderance of evidence from animal or cadaver studies, from conceptual/theoretical models/principles, or from basic science/bench research, or published expert opinion in peer-reviewed journals that supports the recommendation". |
| 53 | British Society for Rheumatology | UK | Creating Clinical Guidelines: British Society for Rheumatology Protocol | <https://www.rheumatology.org.uk/Portals/0/Documents/Guidelines/Guidelines%20Protocol%20edited%20-%20Dec23%20FINAL.pdf?ver=-Kk1kW7Pat7ncErckPrzTw%3d%3d> | 2023 | yes | Consensus recommendation | "Where there is a lack of evidence on a particular question, the GWG should be clear about how a consensus has been reached in formulating a recommendation". |
| 54 | Association of Scientific Medical Societies (AWMF) | Germany | AWMF Guidance Manual and Rules for Guideline Development | <https://www.awmf.org/fileadmin/user_upload/dateien/downloads_regelwerk/awmf-regelwerk-en-2023-v2.1.pdf> | 2023 | yes | Expert consensus | "Not every key question has to be based or even can be based on systematic searches; an answer based on expert consensus is acceptable in justified cases if strong evidence is lacking and a relevant care problem is at stake" |
| 55 | European Society for Radiotherapy and Oncology (ESTRO) | Belgium | European Society for Radiotherapy & Oncology Guidelines Committee Procedures Policy | <https://estropreprod.smartmembership.net/ESTRO/media/ESTRO/Science/Guidelines/Guidelines-Committee-SOP_Sept-2024.pdf> | 2023 | yes | Expert opinion | "Consensus of the panel based on clinical judgement and experience, due to absence of evidence or limitations in evidence" |
| 56 | National Institute for Health and Care Excellence (NICE) | UK | Developing NICE guidelines: the manual | <https://www.nice.org.uk/guidance/pmg20/resources/developing-nice-guidelines-the-manual-pdf-72286708700869> | 2023 | yes | Consensus | "If the committee does not have sufficient evidence to make recommendations in a particular area (for example, if there are gaps in the evidence base or subgroups are under-represented), it may call on external experts (expert witnesses) who can provide additional evidence from their experience and specific expertise, to help the committee to consider and interpret the evidence". |
| 57 | Brazilian Society of Rheumatology | Brazil | Brazilian society of rheumatology methodological guide for the development of evidence‑based clinical guidelines in rheumatology | <https://doi.org/10.1186/s42358-023-00293-4> | 2023 | yes | Clinical experience | "Some health issues are under-explored due to ethical or logistical difficulties, generating low-quality, insufficient, or contradictory evidence. In these scenarios, the development of recommendations can be based on the clinical experience of highly qualified professionals using The Delphi Method". |
| 58 | American Society of Addiction Medicine (ASAM) | US | The ASAM Methodology for Clinical Practice Guidelines | <https://downloads.asam.org/sitefinity-production-blobs/docs/default-source/quality-science/methdology.pdf?sfvrsn=4209f2ae_1> | 2023 | yes | Expert opinion | "Expert-opinion based recommendations when the certainty of evidence for a given question is insufficient". |
| 59 | The Royal Australian and New Zealand College of Obstetricians and Gynaecologists (RANZCOG) | Australia and New Zeeland | RANZCOG Handbook for the development of evidence-based guidelines and statements | <https://ranzcog.edu.au/wp-content/uploads/2022/08/Handbook-for-the-development-of-evidence-based-guidelines.pdf> | 2023 | yes | Consensus recommendation | "Consensus based: where a recommendation is based on clinical opinion and expertise as insufficient evidence available". |
| 60 | European Society of Clinical Microbiology and Infectious Diseases (ESCMID) | Switzerland | ESCMID manual for clinical practice guidelines and other guidance documents | <https://www.escmid.org/fileadmin/escmid/media/pdf/guidelines_journals/Guideline_Manual_2022_04_08_3_version.pdf> | 2024 | yes | Expert opinion | "We understand that there might be a clinical need for recommendations even when published evidence is insufficient. In such cases recommendations should be provided with explicit acknowledgment that they are based on expert opinion. Empirical evidence can be extended to similar interventions based on experts’ opinion using a similar mechanism". |
| 61 | British Society for Haematology (BSH) | UK | BSH Guideline Development Process | <https://b-s-h.org.uk/guidelines/proposing-and-writing-a-new-bsh-guideline> | 2024 | yes | Good practice | "BSH Good Practice Paper (GPP) (~2,000 words) - Used to recommend good practice in areas where there is a less robust evidence-based guidance, but for which a degree of consensus or uniformity is likely to be beneficial to patient care". |
| 62 | Society of American Gastrointestinal and Endoscopic Surgeons (SAGES) | US | SAGES Guidelines Development: Standard OperatingProcedure | <https://www.sages.org/publications/guidelines/guidelines-development-standard-operating-procedure/> | 2024 | yes | Expert opinion | "Expert and consensus opinions still provide value by informing the distillation and interpretation of evidence, without replacing it". |
| 63 | Society for Vascular Medicine (SVM) | US | Manual of Standard Operating Procedures for SVM Publications | <https://www.vascularmed.org/wp-content/uploads/2024/07/CM.SOP-Final-Document-06-19-2024.pdf> | 2024 | yes | Expert consensus | "An expert consensus statement is developed by a panel of multidisciplinary experts utilizing a review of the available data and research gaps to provide evidence and experience-based recommendations that can be applied to clinical practice challenges". |
| 64 | American College of Emergency Physicians (ACEP) | US | ACEP Clinical Policies Development Process | <https://www.acep.org/patient-care/clinical-policies/related-clinical-policy-resources/acep-clinical-policies-development-process> | Accessed March 2024 | yes | Consensus | "Level C recommendations: Recommendations for patient care that are based on evidence from Class of Evidence III studies or, in the absence of any adequate published literature, based on expert consensus. In instances where consensus recommendations are made, “consensus” is placed in parentheses at the end of the recommendation". |
| 65 | U.S. Department of Veterans Affairs | US | The Guideline for Guidelines | <https://www.healthquality.va.gov/documents/GuidelinesForGuidelinesApproved08262022.pdf> | Unclear | yes | Opinion of respected authorities | "Table 1: Level of Evidence (LE) - Opinion of respected authorities, descriptive studies, case reports, and expert committees" |
| 66 | American Cancer Society | US | New American Cancer Society Process for Creating Trustworthy Cancer Screening Guidelines | <https://doi.org/10.1001/jama.2011.1800> | 2011 | unclear | Opinions of expert clinical consultants | / |
| 67 | Faculty of Sexual & Reproductive Healthcare (FSRH) | UK | Faculty of Sexual & Reproductive Healthcare of the Royal College of Obstetrician & Gynaecologists, Clinical Effectiveness Unit Chalmers Centre - Framework for Clinical Guideline Development | <https://fsrh.org/Common/Uploaded%20files/documents/ceu-framework-for-developing-clinical-guidelines-mar-2018-.pdf> | 2018 | unclear | Good Practice Point | / |
| 68 | United European Gastroenterology (UEG) | Austria | UEG framework for the development of high-quality clinical guidelines | <https://doi.org/10.1177/2050640620950854> | 2020 | unclear | / | / |
| 69 | Royal Dutch Society for Physical Therapy | Netherlands | KNGF guideline methodology 2019 | <https://www.kennisplatformfysiotherapie.nl/binaries/content/assets/kennisplatform/onbeveiligd/guidelines/kngf-guideline-methodology-2019.pdf> | 2019 | no | / | / |
| 70 | European Society of Gastrointestinal Endoscopy (ESGE) | Germany | European Society of Gastrointestinal Endoscopy (ESGE) Guideline Development Policy | [http://dx.doi.org/ 10.1055/s-0031-1291747](http://dx.doi.org/%2010.1055/s-0031-1291747) | 2012 | no | / | / |
| 71 | American Gastroenterological Association Institute (AGA) | US | The AGA Institute Process for Developing Clinical Practice Guidelines Part One: Grading the Evidence | <http://dx.doi.org/10.1016/j.cgh.2013.02.001> | 2013 | no | / | / |
| 72 | Clinical Pharmacogenetics Implementation Consortium (CPIC) | US | Incorporation of Pharmacogenomics into Routine Clinical Practice: the Clinical Pharmacogenetics Implementation Consortium (CPIC) Guideline Development Process | <https://doi.org/10.2174/1389200215666140130124910> | 2014 | no | / | / |
| 73 | European Academy of Neurology (EAN) | Austria | Practical recommendations for the process of proposing, planning and writing a neurological management guideline by EAN task forces | <https://doi.org/10.1111/ene.12818> | 2015 | no | / | / |
| 74 | British Association of Dermatologists (BAD) | UK | Updated guidance for writing a British Association of Dermatologists clinical guideline: the adoption of the GRADE methodology 2016 | <https://doi.org/10.1111/bjd.15201> | 2017 | no | / | / |
| 75 | Canadian Critical Care Society (CCCS) | Canada | Canadian Critical Care Society revised process for guideline development and endorsement | <https://doi.org/10.1007/s12630-018-1182-3> | 2018 | no | / | / |
| 76 | American College of Physicians (ACP) | US | The Development of Clinical Guidelines and Guidance Statements by the Clinical Guidelines Committee of the American College of Physicians: Update of Methods | <https://doi.org/10.7326/m18-3290> | 2019 | no | / | / |
| 77 | Canadian Cardiovascular Society (CCS) | Canada | CCS Guidelines Development Procedures and Policies | <https://ccs.ca/app/uploads/2021/07/Gui-development-Proc_policies.pdf> | 2020 | no | / | / |
| 78 | Canadian Association of Gastroenterology (CAG) | Canada | CAG Policy on the Application for, and Implementation of, Clinical Practice Guidelines | <http://cag-acg.com/images/publications/CPG-Guidelines-Policy-23October2020.pdf> | 2020 | no | / | / |
| 79 | College of American Pathologists (CAP) | US | Evidence-based Guideline Development Methodology Manual |  | 2020 | no | / | / |
| 80 | European Dermatology Forum (EDF) | Switzerland | EuroGuiDerm Guideline and Consensus Statement Development Manual | <https://debm.charite.de/fileadmin/user_upload/microsites/ohne_AZ/m_cc12/debm/EuroGuiDe_Manual_v1.3_20-03-03_for_website.pdf> | 2020 | no | / | / |
| 81 | American Thoracic Society (ATS) | US | American Thoracic Society Clinical Practice Guideline Development Manual | <https://www.thoracic.org/statements/document-development/resources/guideline-development-manual.pdf> | 2020 | no | / | / |
| 82 | Estonian Health Insurance Fund | Estonia | Estonian Handbook for Guidelines Development | <https://ravijuhend.ee/uploads/userfiles/Estonian_Handbook_for_Guidelines_Development_2020.pdf> | 2020 | no | / | / |
| 83 | European Respiratory Society (ERS) | Switzerland | ERS Handbook for Clinical Practice Guidelines | <https://ers.box.com/s/fwaabkr604oakr1zfs4egx9suasqzmdu> | 2021 | no | / | / |
| 84 | Society for Cardiovascular Angiography and Interventions (SCAI) | US | SCAI Publications Committee Manual of Standard Operating Procedures: 2022 Update | <https://doi.org/10.1016/j.jscai.2022.100389> | 2022 | no | / | / |
| 85 | International League Against Epilepsy (ILAE) | US | What is a clinical practice guideline? A roadmap to their development. Special report from the Guidelines Task Force of the International League Against Epilepsy | <https://doi.org/10.1111/epi.17312> | 2022 | no | / | / |
| 86 | European Society of Anaesthesiology and Intensive Care (ESAIC) | Belgium | Requirements for development of ESAIC Guidelines. | <https://esaic.org/wp-content/uploads/2024/02/guidelines-production-requirement-for-website-final-sep2022-final.pdf> | 2022 | no | / | / |
| 87 | American Association of Clinical Endocrinology (AACE) | US | Protocol for Development of American Association of Clinical Endocrinology Clinical Practice Guidelines and Consensus Statements: Summary of Methodology, Process, and Policy e 2023 Update | <https://doi.org/10.1016/j.eprac.2023.01.012> | 2023 | no | / | / |
| 88 | Canadian Task Force on Preventive Health Care | Canada | Canadian Task Force on Preventive Health Care Methods Manual | <https://canadiantaskforce.ca/methods/> | 2023 | no | / | / |
| 89 | American Academy of Pediatrics (AAP) | US | [Clinical Practice Guideline Manual](https://www.aap.org/en/advocacy/quality/clinical-practice-guideline-manual/) | <https://www.aap.org/en/advocacy/quality/developing-clinical-recommendations-for-clinical-practice-guidelines/> | 2024 | no | / | / |
| 90 | American Association for the Study of Liver Diseases (AASLD) | US | AASLD Policy on Development and Use ofPractice Guidelines, Guidances, And Position Papers | <https://www.aasld.org/practice-guidelines/aasld-policy-development-and-use-practice-guidelines-guidances-and-position#steps-in-guideline-and-guidance-development-and-approval-process> | Accessed March 2024 | no | / | / |
| 91 | American College of Gastroenterology (ACG) | US | American College of Gastroenterology Practice Parameters Committee - ACG Guideline Creation Process | <https://gi.org/guidelines/> | Accessed March 2024 | no | / | / |
| 92 | Australian National Health and Medical Research Council (NHMRC) | Australia | Guidelines for Guidelines Handbook | <https://www.nhmrc.gov.au/guidelinesforguidelines> | Accessed March 2024 | no | / | / |
| 93 | American Academy of Family Physicians (AAFP) | US | Clinical Practice Guideline Manual | <https://www.aafp.org/family-physician/patient-care/clinical-recommendations/cpg-manual.html> | Accessed March 2024 | no | / | / |
| 94 | American Academy of Sleep Medicine (AASM) | US | AASM Clinical Practice GuidelineDevelopment Process | <https://aasm.org/clinical-resources/practice-standards/guideline-development-process/> | Accessed March 2024 | no | / | / |
| 95 | Swiss Society for Infectious Diseases Society (SSI) | Switzerland | Concept for the development of Clinical Guidelines for the SSI | <https://www.sginf.ch/files/star-guidelines_concept.pdf> | Unclear | no | / | / |
| 96 | Canadian Thoracic Society (CTS) | Canada | CTS Guideline Process At-A-Glance | <https://cts-sct.ca/wp-content/uploads/2020/11/CTS-Guideline-Process-At-A-Glance.pdf> | Unclear | no | / | / |
| 97 | Endocrine Society | US | Endocrine Society Guideline Methodology | <https://www.endocrine.org/-/media/endocrine/files/cpg/methodology-page-refresh/endocrine_society_guideline_methodology_links.pdf> | Unclear | no | / | / |
| 98 | Society for Maternal-Fetal Medicine (SMFM) | US | SMFM Clinical Practice Guidelines Development Process | <https://s3.amazonaws.com/cdn.smfm.org/media/2569/Guidelines_Dev_Process_Public_for_website2.pdf> | Unclear | no | / | / |
