# Supplementary material for: Defining expert opinion in clinical guidelines: insights from 98 scientific societies – a methodological study
Source: BMC Med Res Methodol. 2025 Apr 2;25:87. doi: 10.1186/s12874-025-02534-0 (PMC11963610; doi:10.1186/s12874-025-02534-0)
Supplement: Supplementary file 3 — Additional file 3. EO terminology. [file 12874_2025_2534_MOESM3_ESM.docx]

| **Term** | **No.** | **%** |
| --- | --- | --- |
| Expert opinion | 32 | 49.23 |
| Consensus | 6 | 9.23 |
| Consensus recommendation | 3 | 4.62 |
| Consensus of expert opinion | 3 | 4.62 |
| Expert consensus | 2 | 3.08 |
| Opinion of respected authorities | 2 | 3.08 |
| Good practice statements | 2 | 3.08 |
| Consensus of experts | 1 | 1.54 |
| Consensus based recommendation | 1 | 1.54 |
| Expert consensus statement | 1 | 1.54 |
| Consensus statement | 1 | 1.54 |
| Expert consensus opinion | 1 | 1.54 |
| Best practice | 1 | 1.54 |
| Good practice | 1 | 1.54 |
| Clinical experience | 1 | 1.54 |
| Clinical Practice Information | 1 | 1.54 |
| Expert evidence | 1 | 1.54 |
| Expert-based evidence | 1 | 1.54 |
| Expert Guidance | 1 | 1.54 |
| Inferences from first principles | 1 | 1.54 |
| Opinion | 1 | 1.54 |
| Ungraded Suggestions for Clinical Care | 1 | 1.54 |
|  | 65 | 100.00 |

**Additional file 3 - EO terminology**
